# Supplementary material for: Identification of novel toxins associated with the extracellular contractile injection system using machine learning
Source: Mol Syst Biol. 2024 Jul 28;20(8):859–79. doi: 10.1038/s44320-024-00053-6 (PMC11297309; doi:10.1038/s44320-024-00053-6)
Supplement: Supplementary file 1 — Appendix [file 44320_2024_53_MOESM1_ESM.pdf]

Appendix File 1

Genome-wide discovery of toxins associated with the extracellular contractile injection system

Table of Contents:

Primer Table

Primers name	sequence	Description
EAT14_IP161	gtttttttgggctagcaggaggATGCACACTAACAAGACGGG	Part 1_C298A_fwd_REPEATED
EAT14_IP162	attccaaccgcCTAAAGTGTCTTCACAACAACG	Part 1_C298A_rev
EAT14_IP163	agacactttagGCGGTTGGAATATCCGGTGC	Part 2_C298A_fwd
EAT14_IP164	actctagaggatccccgggtacCTAAAGTGTCTTCACAACAACGCAAAAG	Part 2_C298A_rev_REPEATED
EAT14_IP165	GAACTGGAGCGTGGGGCG	verify mutant EAT14 C298A
EAT14_IP166	tgattagccgcCGCCCACCACATTCCTGAGT	Part 1_H410A_rev
EAT14_IP167	tgtggtgggcgGCGGCTAATCATGCGCACGA	Part 2_H410A_fwd
EAT14_IP168	GACTCAGGAATGTGGTGGGCG	verify mutant EAT14 H410A
EAT14_IP169	tagccgaacgcCGCAAAATTAATGTAGTCCGATC	Part 1_D397A_rev
EAT14_IP170	ttaattttgcgGCGTTCGGCTATTATGATGTTG	Part 2_ D397A_fwd
EAT14_IP171	CGGACTACATTAATTTTGCG	verify mutant EAT14 D397A
EAT15_IP172	gtttttttgggctagcaggaggATGAAGAACAAAAACTATGAGTATGACGTG	Part 1_E83A_fwd
EAT15_IP173	gagacccgtgcTGCGTGATTCGTCCACAGTTTC	Part 1_E83A_rev
EAT15_IP174	cgaatcacgcaGCACGGGTCTCCATGCAAGC	Part 2_ E83A_fwd
EAT15_IP175	actctagaggatccccgggtacCTATGACAATTTATGTTTACAATAATCTATAAGCGCGATTCTCC	Part 2_E83A_rev
EAT15_IP176	CAGAAACTGTGGACGAATCACGCA	verify mutant EAT15 E83A
EAT16_IP177	gtttttttgggctagcaggaggATGACCCAGGGGTTTCCGAC	Part 1- D42A_fwd_REPEAT
EAT16_IP178	tcgatcccaaaGGCATGCTCTTGATGATTACGC	Part 1- D42A_rev
EAT16_IP179	aagagcatgccTTTGGGATCGATGCCCAAGTAG	Part 2- D42A_fwd
EAT16_IP180	actctagaggatccccgggtacTTATGCATCGTCCGTCGGAAG	Part 2- D42A_rev_REPEAT
EAT16_IP181	tctacttgggcGGCGATCCCAAAATCATGCTC	Part 1- D46A_rev

EAT16_IP182	ttgggatcgccGCCCAAGTAGACGTTATCCTG	Part 2- D46A_fwd
EAT16_IP183	gaagaaccgtaGGCTATCTGCAGCGCCAGCA	Part 1- K68A_rev
EAT16_IP184	tgcagatagccTACGGTTCTTCATTCTTCACCG	Part 2- K68A_fwd
