# Supplementary figures and images for: Identification of novel toxins associated with the extracellular contractile injection system using machine learning
Source: Mol Syst Biol. 2024 Jul 28;20(8):859–79. doi: 10.1038/s44320-024-00053-6 (PMC11297309; doi:10.1038/s44320-024-00053-6)

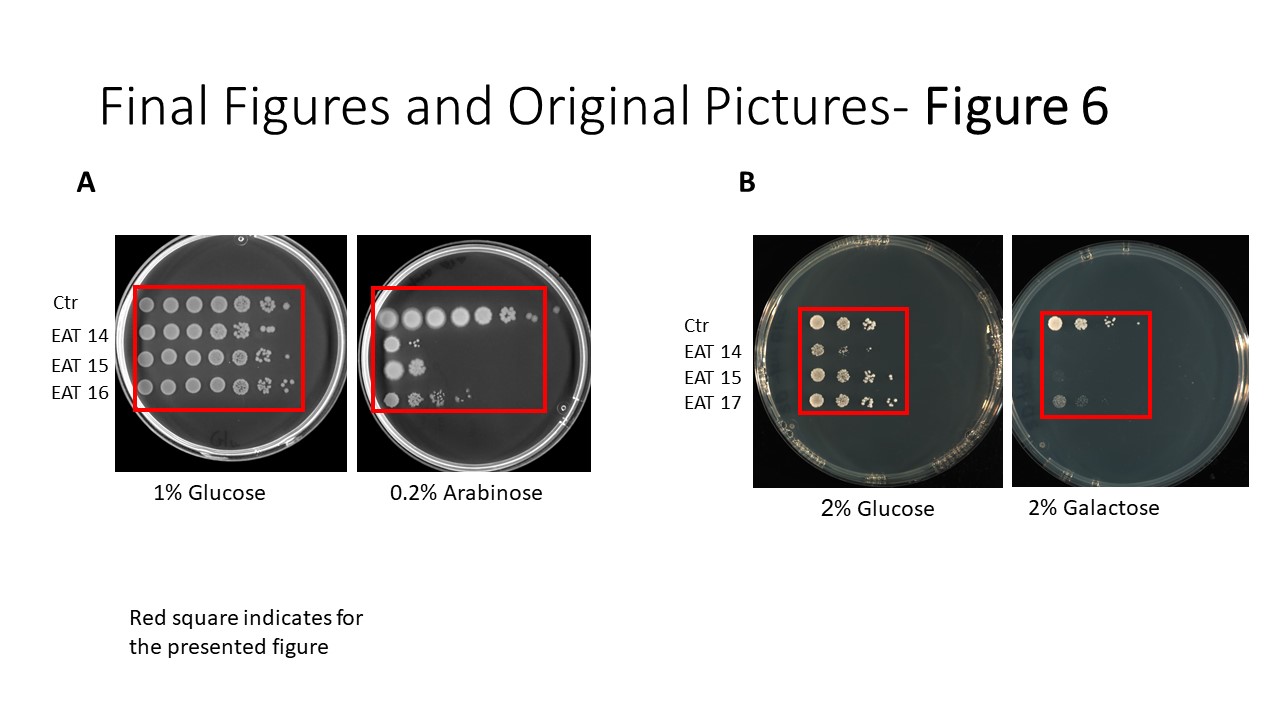

Supplement: Supplementary file 7 — Source data Fig. 6 [file 44320_2024_53_MOESM7_ESM.zip › Fig 6A-B/fig 6 source data.jpg]

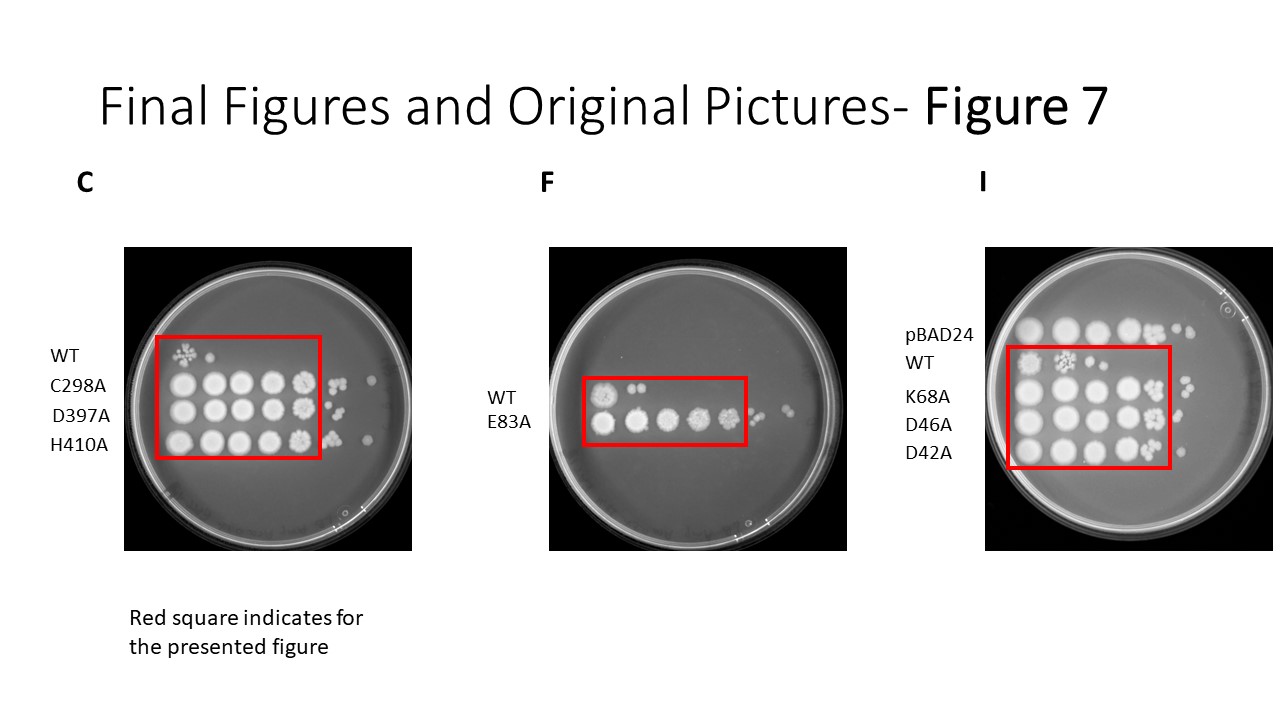

Supplement: Supplementary file 8 — Source data Fig. 7 [file 44320_2024_53_MOESM8_ESM.zip › Fig 7C, F, I/fig 7 source data.jpg]
