# Supplementary material for: Identification of novel toxins associated with the extracellular contractile injection system using machine learning
Source: Mol Syst Biol. 2024 Jul 28;20(8):859–79. doi: 10.1038/s44320-024-00053-6 (PMC11297309; doi:10.1038/s44320-024-00053-6)
Supplement: Supplementary file 10 — Expanded View Figures [file 44320_2024_53_MOESM10_ESM.pdf]

Expanded View Figures

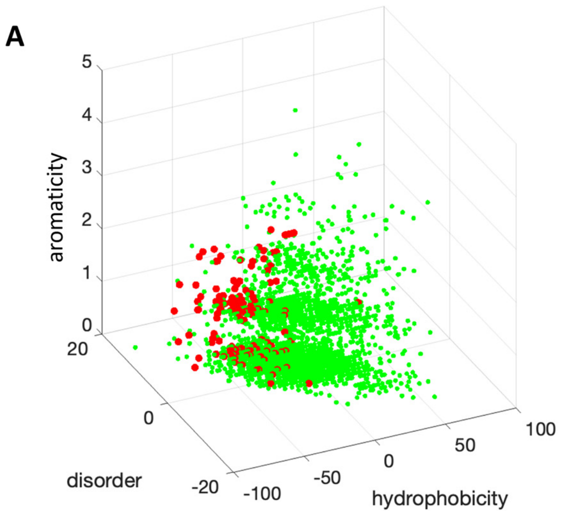

Figure EV1. The scatterplot of three sequence-based features that were used to encode eCIS signal peptide score based on linear SVM.

(**A**) The red dots are EATs and the green dots are non-EATs. (**B**) The colormap is defined by the score values with red being high values and yellow being near zero values. The plots show the high consistency between the positive set and the higher score values.

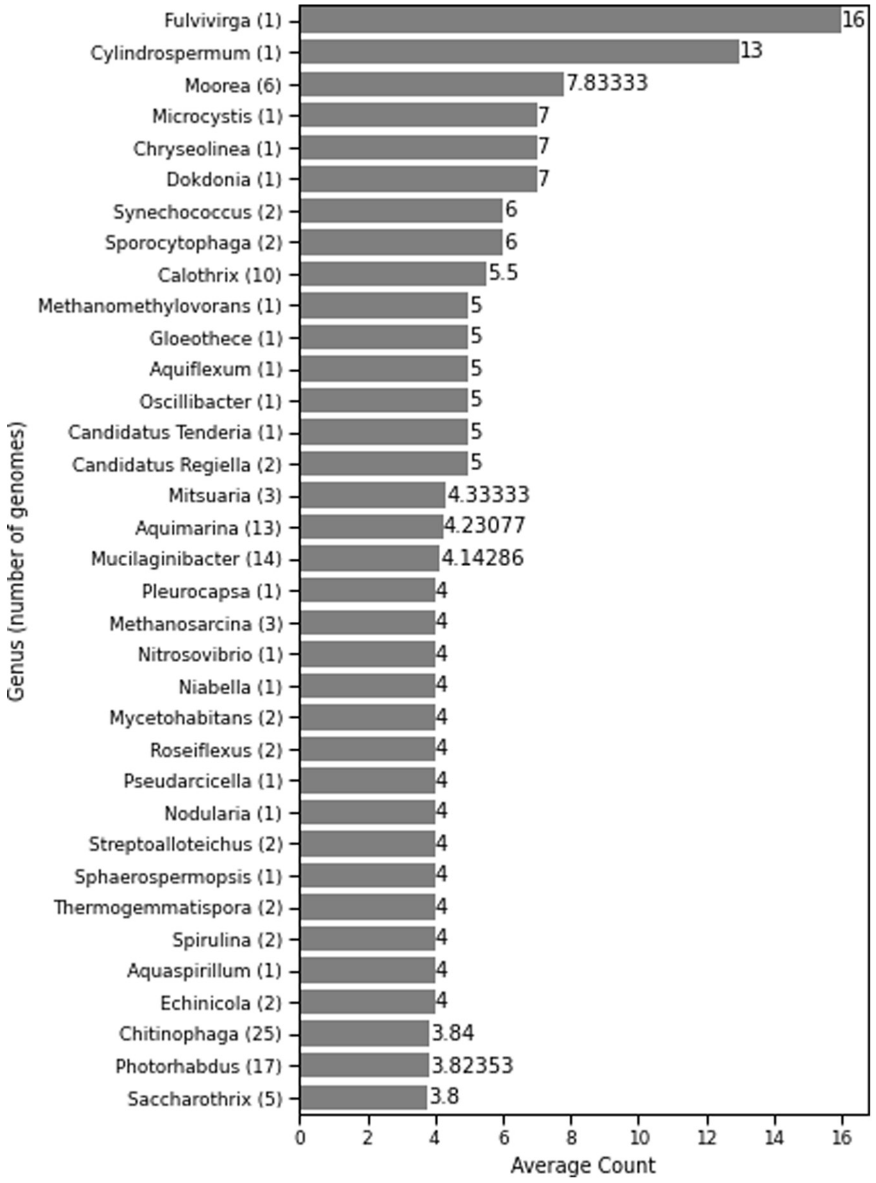

Figure EV2. Average number of predicted EATs per genus.

In parenthesis is the number of genomes per genus in our dataset.

© The Author(s)

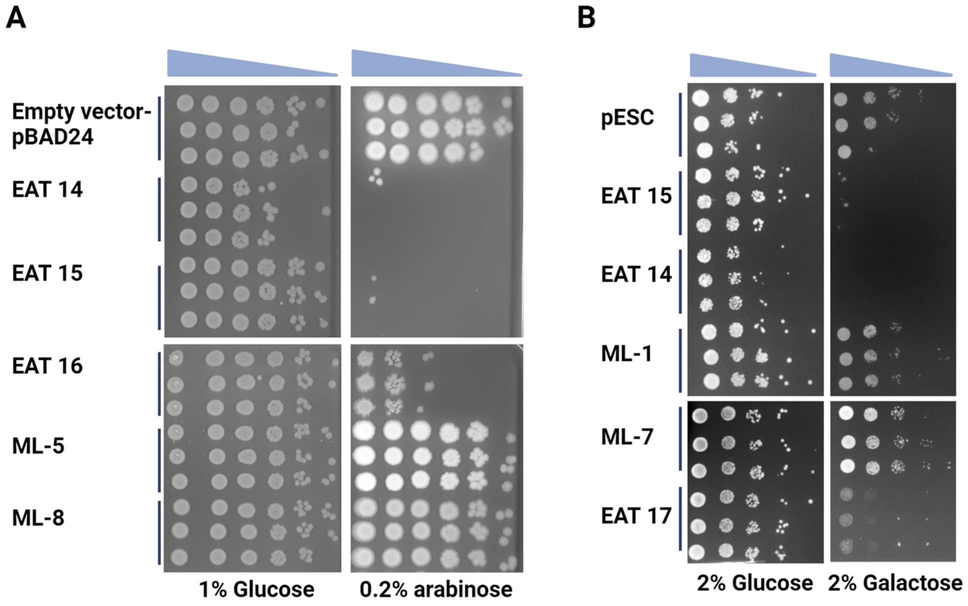

Figure EV3. Drop assays including the predicted effectors that were not toxic.

(**A**) Bacterial drop assay. Induced with 0.2% Arabinose and uninduced in the presence of 1% Glucose. Light blue triangles indicate the serial dilution of the droplets. (**B**) Yeast drop assay, induced with 2% Galactose and inhibited with 2% Glucose. For the negative control in the yeast drop assay, a pESC empty vector was used. Candidates who did not work maintained the annotation ML-X (ML- preadicted by Machine Learning).
